# Academic libraries and collaborative research services

**DOI:** 10.29173/jchla29644

**Published:** 2022-12-01

**Authors:** Debbie Chaves

**Affiliations:** Head, Copyright and Course Resources Wilfrid Laurier University 75 University Avenue Waterloo, ON N2L 3C5

Forbes, C. **Academic libraries and collaborative research services**. London, U.K.: Rowman & Littlefield; 2022. Softcover: 312 pages. ISBN: 9781538153697. Price: USD$55.00. Available from: https://rowman.com/ISBN/9781538153697/Academic-Libraries-and-Collaborative-Research-Services.

A constant question for academic librarians is how to create an organizational structure and collaborate with faculty in a way that meets the future research demands of the academy. *Academic libraries and collaborative research services* addresses this issue without centring on the liaison librarian role. Instead, it focuses on case studies of how academic libraries connect with research services on campus. The book’s editor, Carrie Forbes, is a Professor and Associate Dean at the University of Denver Libraries, and coeditor of *Rethinking reference for academic libraries: innovative developments and future trends* (2014). This newest book is timely, informative, and well researched. The writing quality is consistent throughout the collection of case studies of American libraries, despite being written by various authors.

The book is organized into four parts, including: (1) emerging liaison roles, (2) research data services, (3) publishing, and (4) professional development. The opening chapters address the new types of roles that liaison librarians participate in and to what degree the university community understands the roles of scholarly communication librarians. Unsurprisingly, the new roles are just technological extensions of liaison librarians' old roles of connecting the library’s resources with patrons. Most of the university community does not understand what these roles are. There are always university-wide stakeholders who engage with librarians regularly and are heavy users of library resources, and then there are many more who do not understand what librarians actually do. One central role of librarians is outreach, and these chapters offer some solutions to the problem of how to inform the academy about the nature of library work.

Health librarians would be most interested in Gregory Laynor and Stephanie Roth’s chapter, entitled Librarians as research partners for developing synthesis protocols. The authors use the term “evidence synthesis” and not “knowledge synthesis” to represent the landscape of systematic and scoping reviews. They see the role of librarians as being essential due to the inclusion of protocol registration and centre their service around it. Peer review of search strategies as described by PRESS is included in the service, but the authors do not address a mechanism of search strategy peer review for groups of libraries and librarians. The role of authorship is addressed with advice to include the conversation early in the protocol process. The authors suggest a fee-based structure to assist libraries financially in the role of protocol creation for evidence synthesis. However, monetizing evidence synthesis work would not be feasible for librarians who work in unionized faculty environments. A list of evidence synthesis protocol resources is provided at the end of the chapter, which is very helpful.

The section on data is short and addresses very unique case studies, including one about offering data services and another about using RedCap for data management. The Canadian data management climate is different from the American data management system. In Canada, there is a national framework for the management of data and a program to support it in Borealis.

The chapters addressing digital humanities were institutionally specific but well-written and had interesting uses for varying technologies. Even though this is a section dealing specifically with open access issues, the editor does not have an ORCID (an Open Researcher and Contributor ID). A chapter on persistent identifiers (PIDs) like ORCID would have been valuable. The more tools that academic librarians can have in their toolkit, the more opportunity they will have to open the conversation with faculty regarding research collaboration.

The professional development section looked at emerging leadership, interpersonal skills, and data. Additions to this section could have included addressing specific mechanisms to obtain time and capacity for professional development as well as artificial intelligence in academic libraries. However, the chapter on tips, strategies, and methods to promote and enhance interpersonal skills was interesting and thoughtful.

The authors focus primarily on American academic libraries with only one chapter looking elsewhere (a liaison collaboration with students in France and Morocco). How universities are organized in the United States can be structurally different from libraries in Canada, Australia, and the United Kingdom. While Canadian academic libraries make a concerted effort to create and maintain a relationship with their research offices, other countries have different relationships between the library and the research office. I would have assumed that this volume would spend more time discussing this relationship as key to how the academic library collaborates with research services on campus. However, only chapter 12, Leveraging research information management to center the library as campus leader, addresses the Research Information Management (RIM) system (called the Current Information System (CRIS) in the U.K.), and how libraries assist the research office. The authors do not address training and staffing concerns for this relationship, which raises questions such as: Who helps the research office? Who does the bibliometric analysis? Who manages the RIM? What work would an academic library have to stop doing to assist in these endeavours? In addition, by focusing only on the U.S. context, the book overlooks other ways for academic libraries to forge a relationship with research offices. Structural differences are important for Canadian academic libraries to acknowledge, as the U.S. can run a very different show and we do not have to emulate them.

I would recommend this book as I see it as a timely addition to conversations around the practice of academic librarianship. It is useful for both academic librarians and health science librarians looking to understand their role in the academic library environment. The book is not as expensive as books published through the American Libraries Association (ALA). If you were looking for something that focuses on liaison librarian models specifically, I would also recommend *Approaches to liaison librarianship: innovations in organization and engagement* (Canuel and Crichton 2021).
